# Snake Envenomation Causing Distant Tracheal Myonecrosis

**DOI:** 10.1155/2013/364195

**Published:** 2013-09-05

**Authors:** Amina Khimani, Afton Mcnierney, Sara Surani, Salim Surani

**Affiliations:** ^1^University of California, Berkeley, CA 94704, USA; ^2^Christus Spohn Hospital Emergency Medicine Residency, Corpus Christi, TX 78404, USA; ^3^Pulmonary Associates, Corpus Christi, TX 78413, USA; ^4^Texas A&M University, 1177 W Wheeler Avenue, Suite 1, Aransas Pass, TX 78336, USA

## Abstract

Snakebites are often believed to be poisonous. However, this is not always the case. In fact, each bite differs from snake to snake, depending on if the snake is poisonous and if there is envenomation. Venom in pit viper snakebites is often associated with local necrosis. The abundant literature selections and research articles justify local myonecrosis due to envenomation, but there is not much in the literature regarding myonecrosis at a site distant from the snakebite. We hereby present a case of a 42-year-old man who was transferred to our emergency department after a rattlesnake bit him twice. The patient, besides developing local myonecrosis at the site of the snakebite, developed necrosis of the scrotum as well as tracheal pressure myonecrosis at the site of the endotracheal tube balloon. In this review, we will attempt to discuss the myonecrosis pathophysiology and management related to the rattle snakebite.

## 1. Introduction

Out of the 3000 snake species found worldwide, only about 15% are dangerous to the human population [1]. In the United States specifically, approximately 20 of the 120 native snakes are venomous [1]. Snake venom can be classified into five different categories: hemotoxic, neurotoxic, necrotoxic, cardiotoxic, and nephrotoxic. The predominant effect depends on which family the snake belongs to. The degrees of intoxication due to envenomation are described in Table 1 [2].

Envenomation can lead to different levels of intoxication, depending on several factors: snake size, species, amount of venom injected, location of bite, treatments provided, timing of treatments, and previous medical history [1]. When examining the pit viper snake exclusively, the following results may occur due to envenomation: puncture wounds, pain, ecchymosis, lymphangitis, hemorrhagic bullae, and necrosis (or tissue destruction) [3]. Local necrosis due to viper bites usually appears to be ischemic and develops slowly over weeks, presenting like dry gangrene [4].

## 2. Case Presentation

**Figure 1:**
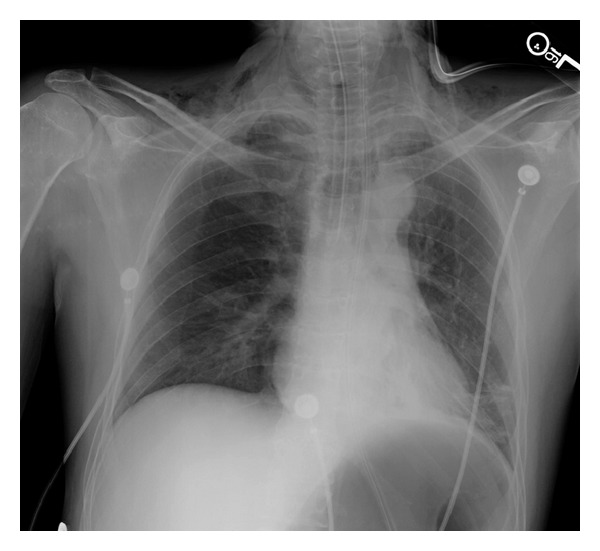
X-ray chest AP view showing endotracheal tube in the right mainstem carina.

**Figure 2:**
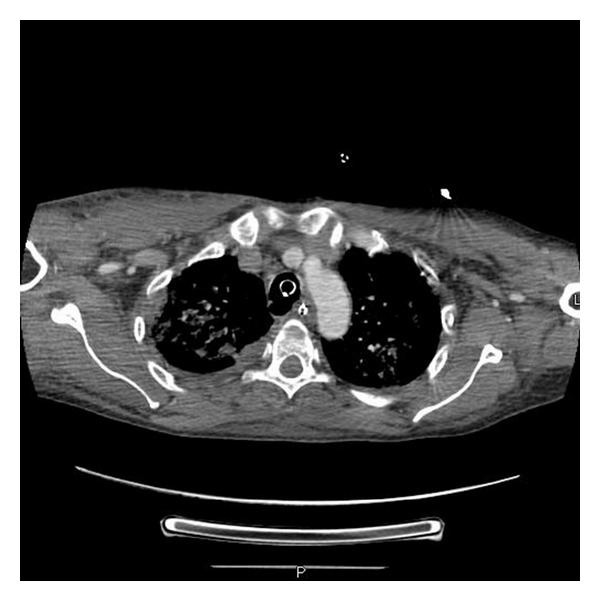
CT scan of chest mediastinal view showing endotracheal tube in trachea and also showing weakness in the posterior membranous wall of trachea.

**Figure 3:**
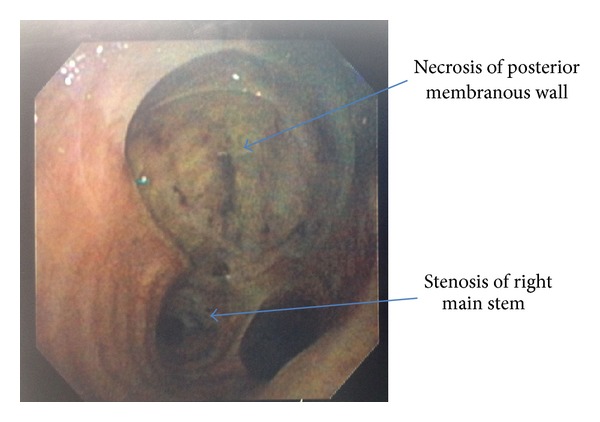
Bronchoscopy image showing necrosis of posterior membranous wall just above main stem carina.

A 42-year-old male, reportedly at a bonfire on the beach, was bitten by a rattlesnake twice, allowing for double envenomation. He was taken to the outlying facility where he was in anaphylactic shock, was intubated, and was started on vasopressor therapy with norepinephrine. The patient received six vials of Crotalidae Polyvalent Immune Fab (Ovine) (CroFab) and was transferred to our facility on a ventilator. Patient WBC was 13.1/L, hemoglobin 16 gm/dL, hematocrit 49.8%, and platelet count 279,000/L. Patient blood glucose was 279 mg/dL. Other chemistries were within normal limits. Patient's creatine kinase level (CK) was 768/L with normal troponin T level. The patient's endotracheal tube (ET) was found to be in the right main stem carina (Figure 1). ET tube was pulled back by 2 cm within a few hours of the right main stem intubation. The patient received 100 vials of CroFab. He also went into disseminated intravascular coagulation (DIC) with platelet count dropping down to 24 K and D-dimer 16.4 ng/mL, and fibrinogen degradation product was >40 mg/mL. The patient was supported with fresh frozen plasma and blood products. He also developed severe skin necrosis at the site of the bite. Due to the worsening respiratory status and declining oxygen status, a CT scan of the chest was done, which, besides revealing pneumonia, revealed bulging of the posterior membranous tracheal wall just above the right main stem carina, where the ET tube was before being pulled upward (Figure 2). The patient underwent a bronchoscopy for pneumonia and respiratory failure. The bronchoscopy revealed tracheal pressure myonecrosis of the membranous portion of the trachea, just above the right main stem carina, and narrowing of the opening of the right main stem carina and right minor carina due to bronchial wall inflammation (Figure 3). Local necrosis due to snake venom is a common presentation after snakebite. Distant pressure necrosis has not been reported. We hereby present a case of tracheal necrosis distant to the site of envenomation. The patient responded to the therapy, and a repeat bronchoscopy showed healing of the membranous trachea and a resolution of the stenosis of the right main stem.

## 3. Discussion

Out of the fifteen total snake families, only about four are known to be poisonous: colubrids, elapids, vipers, and sea snakes. These snake venoms are made of small molecules such as adrenaline, serotonin, and histamine, along with a large number of biologically active polypeptides [5]. Viper venom, specifically, has effects locally, as well as systemically. Locally, there is rapid swelling and necrosis in 5–10% of patients, caused by some vipers only. Systemically, there are vasculotoxic effects such as abnormal bleeding, nonclotting blood, with some vipers only, and shock. However, there is one exception within the viper family: the rattlesnake, *Crotalus durissus*. This particular rattlesnake's venom has mainly neurotoxic effects, rather than vasculotoxic like other vipers [4]. Neurotoxic effects are comprised of ptosis, respiratory paresis, glossopharyngeal palsy, and cardiac effects. When examining the side effect of myonecrosis, there are only three toxins capable of causing this damage: phospholipases, cardiotoxins, and myotoxins. Rattlesnake venom falls under the category of phospholipases [5].

When examining pit vipers in North America, local necrosis is often a feature of envenomation. Signs of muscle necrosis in human victims are muscle pain, physical weakness, elevated plasma CK, and aspartate aminotransferase levels (AST) [6]. Treatment for envenomation includes cleaning the wounds and administering tetanus toxoid, or tetanus immune globulin. Patients should also be given intravenous fluid, and blood must be drawn from an extremity that is unaffected. The leading edge of the swelling should be marked and measured every thirty minutes. Anti-snake venom, ideally, should also be administered within 4 hours of the bite but can still be effective within 24 hours. Anti-snake venom is made by immunizing large animals, like horses, with venom or multiple venoms. Then, serum from the animal is processed by salt, or acrylic acid and an enzyme, papain, or pepsin. The main purpose of antivenom is to neutralize circulating venom, eliminating the toxic effects [7].

To save the life of a patient with bulbar and respiratory paralysis, antivenom treatment alone is not the answer. Endotracheal intubation and mechanical ventilation must occur once respiratory distress, loss of gag reflux, or failure to cough occurs. Because many snakes have aerobic and anaerobic bacteria in their mouths, a single dose of antibiotic course, a prophylactic course of penicillin, along with a booster dose of tetanus toxoid is suggested [6]. Fasciotomy is not recommended as a prophylactic intervention and should only be done if compartment pressure exceeds 30 mm Hg, which needs to be serially monitored [8].

In our patient, beside local myonecrosis, distant tracheal myonecrosis was observed as well. Studies have shown no evidence of distant tracheal myonecrosis occurring in rattlesnake envenomation per our review of the literature. In our case, we felt that the tracheal myonecrosis may have happened because of the ET cuff pressure causing necrosis of the posterior membranous wall of the trachea, which may have been weakened by the high dose of snake venom.

## 4. Conclusion

Generally, local myonecrosis occurs once a venomous bite from a pit viper strikes. However, in this case, the patient had envenomation that occurs twice in a short period of time and developed myonecrosis local as well as distant to the site of the bite. Our patient shows that we should be wary of heavy envenomation, as it may even lead to distant myonecrosis.

## Figures and Tables

**Table 1:** Degree of intoxication due to envenomation.

Degree of intoxication	Symptoms
1 (no intoxication)	Dry bite, indicating no intoxication
2 (mild intoxication)	Local edema and pain
3 (moderate intoxication)	Pain, systemic signs, and edema spreading beyond bite zone
4 (severe intoxication)	Shock, massive edemas, and severe coagulopathy
